# Bacterial coinfections in hospitalized children with COVID-19 during the SARS-CoV-2 Omicron BA.2 variant pandemic in Taiwan

**DOI:** 10.3389/fmed.2023.1178041

**Published:** 2023-04-18

**Authors:** Huan-Cheng Lai, Yu-Lung Hsu, Chien-Heng Lin, Hsiu-Mei Wei, Jiun-An Chen, Yan-Yi Low, Yu-Ting Chiu, Hsiao-Chuan Lin, Kao-Pin Hwang

**Affiliations:** ^1^Division of Pediatric Infectious Diseases, China Medical University Children’s Hospital, China Medical University, Taichung, Taiwan; ^2^School of Medicine, College of Medicine, China Medical University, Taichung, Taiwan; ^3^Division of Pediatric Pulmonology, China Medical University Children’s Hospital, China Medical University, Taichung, Taiwan; ^4^Department of Biomedical Imaging and Radiological Science, College of Medicine, China Medical University, Taichung, Taiwan

**Keywords:** bacterial infection, children, coinfection, COVID-19, hospitalization

## Abstract

**Background:**

Bacterial coinfections have been widely recognized in adults with coronavirus disease 2019 (COVID-19). However, bacterial coinfections in hospitalized children with severe acute respiratory syndrome coronavirus 2 (SARS-CoV-2) have not been sufficiently researched. This study aimed to determine the clinical presentations and risk factors for bacterial coinfections of pediatric inpatients during the SARS-CoV-2 Omicron BA.2 variant pandemic.

**Methods:**

This retrospective, observational study included patients younger than 18 years of age who were hospitalized for COVID-19 confirmed by polymerase chain reaction (PCR) or antigen rapid tests during the SARS-CoV-2 Omicron BA.2 variant pandemic. Data and outcomes of these patients with or without bacterial coinfections were compared.

**Results:**

During this study period, 161 children with confirmed COVID-19 were hospitalized. Twenty-four had bacterial coinfections. The most frequently reported concurrent diagnosis was bacterial enteritis, followed by lower respiratory tract infections. Children with bacterial coinfections had higher white blood cell (WBC) counts and PCR cycle threshold values. The bacterial coinfection group comprised a relatively greater proportion of patients who required high-flow nasal cannula oxygen and remdesivir. The length of stay in the hospital and that in the intensive care unit were longer for children with COVID-19 with bacterial coinfections. Mortality was not observed in either group. Abdominal pain, diarrhea, and comorbidity with neurologic illnesses were risk factors for bacterial coinfections with COVID-19.

**Conclusion:**

This study provides clinicians with reference points for the detection of COVID-19 in children and its possible association with bacterial infections. Children with COVID-19 and neurologic diseases who present with abdominal pain or diarrhea are at risk of bacterial coinfections. Prolonged fever duration and higher PCR test cycle threshold values, WBC levels, and high-sensitivity C-reactive protein (hsCRP) levels may indicate bacterial coinfections in children with COVID-19.

## Introduction

Bacterial coinfections can occur with respiratory tract infections. Before severe acute respiratory syndrome coronavirus 2 (SARS-CoV-2) was discovered, the influenza virus was a well-established respiratory pathogen that predisposed patients to the development of bacterial infections. Bacterial coinfections with influenza viruses can cause progression to pneumonia or pulmonary empyema and lead to bacteremia or sepsis in children (1). Bacterial coinfections with influenza are also associated with more serious diseases and worse consequences for children (2). *Streptococcus pneumoniae*, *Haemophilus influenzae*, and *Staphylococcus aureus* are the most common causes of bacterial infections for influenza patients (2–4). However, different respiratory viruses may present with different types of bacterial coinfections and have various clinical presentations and outcomes.

In November 2021, the World Health Organization classified the SARS-CoV-2 Omicron variant as a variant of concern. The rapid spread of the Omicron variant resulted in a global pandemic that quickly displaced the Delta variant (5). In Taiwan, Omicron expanded rapidly, resulting in a pandemic in April 2022 (6). After this surge, children were more likely to be infected with the Omicron variant than with the previous SARS-CoV-2 variant. Increasing rates of hospitalization for pediatric coronavirus disease 2019 (COVID-19) were reported during the Omicron variant pandemic, especially among children 0 to 4 years of age (7, 8). Among symptomatic patients with infection caused by Omicron, fever and symptoms related to upper airway inflammation are most commonly reported (9). However, it is difficult to clinically determine the presence of bacterial coinfections. Children with COVID-19 experienced less serious illness with Omicron than adults; furthermore, they experienced less serious illness with Omicron than with previous variants (7, 8, 10). However, bacterial coinfections in children with COVID-19 may increase mortality and morbidity rates.

Although bacterial coinfections have been observed in adults infected with previous SARS-CoV-2 variants, studies specifically focused on the epidemiology of COVID-19-associated bacterial coinfections with Omicron variants are lacking. Furthermore, bacterial coinfections among children infected with the Omicron variant are not clear. This study aimed to examine the spectrum of bacterial coinfections in hospitalized children with COVID-19. Pediatric patients admitted to a tertiary medical center during the Omicron outbreak were investigated to identify clinical characteristics and laboratory parameters that could predict the presence of bacterial coinfections.

## Materials and methods

This retrospective, observational study was conducted at China Medical University Children’s Hospital, which is a tertiary medical center in central Taiwan. The study period extended from 1 May 2022 to 4 July 2022. According to the Taiwan Centers for Disease Control, this period comprised the SARS-CoV-2 Omicron BA.2 pandemic, and no other SARS-CoV-2 variants were detected in indigenous patients. Children younger than 18 years of age with COVID-19 confirmed by polymerase chain reaction (PCR) or antigen rapid tests admitted to China Medical University Children’s Hospital were enrolled in this study. All patients were admitted via the emergency department according to the Taiwan Centers for Disease Control infection control policy.

Demographics, clinical manifestations, laboratory data, medical management, and outcomes were obtained from the electronic medical records. Children with COVID-19 with bacterial coinfections were categorized as the confirmed and probable bacterial coinfection group. We defined confirmed bacterial infections as microbiologically proven using blood, urine, stool, or cerebrospinal fluid culture tests and using multiplex gastrointestinal PCR test results indicating bacteria from the stool. Probable bacterial infections were defined as radiographic findings suggestive of a bacterial infection, laboratory parameters compatible with or suggestive of a bacterial infection, and clinical diagnosis by an expert. Collecting respiratory specimens from children can be challenging, and sputum coughed out by children may not accurately represent the pathogenic bacteria of the lower respiratory tract. Therefore, probable lower respiratory tract bacterial infections such as pneumonia and bronchopneumonia were not only diagnosed according to chest X-ray but also clinical symptoms, physical examination, laboratory data, and considering the presence of comorbidities. All patients with probable bacterial infections received antibiotic treatment and were supervised by pediatric infectious specialists.

Descriptive statistics are presented as the median (interquartile range) for continuous variables and as the number and proportion (%) for categorical variables. We compared the clinical characteristics of children with COVID-19 with and without bacterial coinfections using the Mann–Whitney U test for continuous variables or the chi-square test and Fisher’s exact test for categorical variables. To minimize the likelihood of a type I error caused by multiple comparisons when examining symptoms between the two groups, we employed the Bonferroni correction procedure. Significance was established at an adjusted *P*-value of < 0.003 for the comparison of symptoms. Univariable and multivariable logistic regression were established to determine the risk factors for bacterial coinfections in children with COVID-19. Variables with *P* ≤ 0.20 in the univariable analyses were included in the multivariable logistic regression analysis, and the enter method was used to determine baseline risk factors. All analyses were performed using MedCalc^®^ Statistical Software (version 20.118; MedCalc Software Ltd., Ostend, Belgium; 2022).^1^ *P* < 0.05 was considered statistically significant. All tests were two-tailed.

This study was approved by the Institutional Review Board of China Medical University Children’s Hospital (CMUH111-REC3-134). Informed consent was waived. All methods of this study were performed in accordance with the relevant guidelines and regulations.

## Results

**FIGURE 1:**
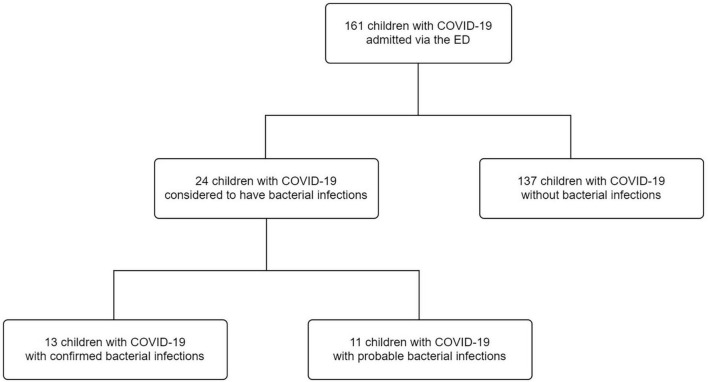
Flow diagram of the study participants. COVID-19, coronavirus disease 2019; ED, emergency department.

One hundred sixty-one children with confirmed COVID-19 were admitted to China Medical University Children’s Hospital. Of these, 24 children (14.9%) were diagnosed with bacterial infections, 13 children (8.1%) had confirmed bacterial infections, and 11 children (6.8%) had probable bacterial infections (Figure 1). Among the 13 children with confirmed bacterial infections, 6 (46.2%) were diagnosed with bacterial enteritis, 3 (23.1%) were diagnosed with acute pyelonephritis, 1 (7.7%) was diagnosed with renal abscess, 1 (7.7%) was diagnosed with bacteremia sepsis, 1 (7.7%) was diagnosed with cellulitis and abscess formation, and 1 (7.7%) was diagnosed with acute purulent otitis media (Supplementary Table 1). *Salmonella* spp. (*n* = 2), *Campylobacter* spp. (*n* = 2), and *Clostridium difficile* (*n* = 3) were detected using a stool culture or multiplex PCR test. The urine culture isolates were *Escherichia coli* (*n* = 4). One child had positive blood culture result. The blood culture isolate was *E. coli*. Methicillin-sensitive and methicillin-resistant *S. aureus* were found in the pus of children with cellulitis and otitis media, respectively (Supplementary Table 1).

There were no differences in the distributions of age, sex, peak body temperature, and duration of fever before admission (Table 1). The cycle threshold value of the PCR test was significantly higher for children with bacterial coinfections [16.6 (13.3–24.8) vs. 13.2 (12.1–17.7); *p* = 0.043]. Compared with children with only COVID-19, a significantly higher percentage of children with COVID-19 with bacterial coinfections presented with abdominal pain (33.3 vs. 2.2%; *p* < 0.001) and diarrhea (41.7 vs. 9.5%; *p* < 0.001). The percentage of comorbidity with the neurologic disease was higher in children with bacterial coinfections (12.5 vs. 0.7%; *p* = 0.007) (Table 1).

**TABLE 1:** Comparison of demographic profiles, clinical features, and comorbidities of both groups.

Variable median (IQR or %)	COVID-19 with bacterial coinfection (*N* = 24)	COVID-19 (*N* = 137)	*p*-value
Age, years	2.0 (0.5–4.0)	1.0 (0.0–3.0)	0.102
Sex, male	14 (58.3)	79 (57.7)	0.951
Confirmed with PCR	21 (87.5)	81 (59.1)	**0.008**
PCR CT value	16.6 (13.3–24.8)	13.2 (12.1–17.7)	**0.043**
Fever	22 (91.7)	134/137 (97.8)	0.336
Fever, °C	39.8 (39.0–40.0)	39.3 (38.9–40.0)	0.193
Fever duration before admission, days	1.0 (0.8–3.3)	1.0 (0.0–2.0)	0.075
**Symptoms**			
Cough	16 (66.7)	93 (69.4)	0.790
Rhinorrhea	10 (41.7)	58 (42.3)	0.951
Sore throat	2 (8.3)	10 (7.3)	0.808
Respiratory distress	2 (8.3)	22 (16.1)	0.503
Wheezing	0 (0.0)	1 (0.7)	0.323
Stridor	0 (0.0)	19 (13.9)	0.110
Abdominal pain	8 (33.3)	3 (2.2)	**< 0.001**
Vomiting	2 (8.3)	16 (11.7)	0.898
Diarrhea	10 (41.7)	13 (9.5)	**< 0.001**
Decreased intake	10 (41.7)	71 (51.8)	0.359
Decreased activity	8 (33.3)	54 (39.4)	0.572
Neurologic symptoms	2 (8.3)	29 (21.2)	0.234
Myalgia	0 (0.0)	10 (7.3)	0.364
Skin rash	1 (4.2)	3 (2.2)	0.891
**Comorbidity**			
Neurologic disease^1^	3 (12.5)	1 (0.7)	**0.007**
Malignancy^2^	0 (0.0)	1 (0.7)	0.323
Chronic lung disease^3^	1 (4.2)	1 (0.7)	0.687
Chronic kidney disease^4^	0 (0.0)	1 (0.7)	0.323
Endocrine disease^5^	0 (0.0)	2 (1.5)	0.687
Rheumatic disease^6^	0 (0.0)	1 (0.7)	0.323

^1^Neurologic disease: epilepsy under medication control*1, Duchenne muscular dystrophy*1, spastic quadriplegic cerebral palsy*1, Dravet syndrome*1.

^2^Malignancy: acute lymphoblastic leukemia*1.

^3^Chronic lung disease: bronchopulmonary dysplasia*1, lung fibrosis with chronic ventilator use*1.

^4^Chronic kidney disease: chronic renal failure s/p renal transplantation*1.

^5^Endocrine disease: phenylketonuria*1.

^6^Rheumatic disease: systemic lupus erythematosus*1.

COVID-19, coronavirus disease 2019; CT, cycle threshold; IQR, interquartile range; PCR, polymerase chain reaction.

Bold values indicate that the *p*-value < 0.05.

Children with COVID-19 had significantly lower white blood cell (WBC) levels than children with COVID-19 and bacterial coinfections [6700.0 (4900.0–9700.0) vs. 8950.0 (6,500.0–10,900.0); *p* = 0.020]. The high-sensitivity C-reactive protein (hsCRP) level was higher in the bacterial coinfection group; however, it was not statistically significant [0.8 (0.0–3.2) vs. 0.3 (0.1–1.0); *p* = 0.078] (Table 2). A relatively higher proportion of children in the bacterial coinfection group required high-flow nasal cannula oxygen (16.7 vs. 2.9%; *p* = 0.019) and remdesivir treatment (12.5 vs. 1.5%; *p* = 0.025). The length of the hospital stay and that of the intensive care unit stay were longer for children with COVID-19 with bacterial coinfections. No mortality occurred in the two groups (Table 3).

**TABLE 2:** Comparison of laboratory parameters of both groups.

Variable median (IQR)	COVID-19 with bacterial coinfection (*N* = 24)	COVID-19 (*N* = 137)	*p*-value
WBC count, per μL	8,950.0 (6,500.0–10,900.0)	6,700.0 (4,900.0–9,700.0)	**0.020**
Neutrophils, %	54.6 (39.5–72.1)	55.8 (37.6–72.7)	0.778
Lymphocytes, %	26.5 (16.2–32.5)	31.0 (14.4–46.4)	0.572
Neutrophil/lymphocyte ratio	1.9 (1.5–4.5)	1.7 (0.8–5.1)	0.637
Monocyte, %	10.4 (7.9–16.7)	11.7 (8.7–15.4)	0.803
Eosinophil, %	0.4 (0.1–2.0)	0.5 (0.1–1.5)	0.819
Basophil, %	0.4 (0.2–0.6)	0.4 (0.2–0.6)	0.769
Band, %	0.0 (0.0–0.0)	0.0 (0.0–0.0)	0.278
Hb, g/dL	11.5 (11.0–12.9)	12.0 (11.3–12.8)	0.668
Platelets, per μL	280,000.0 (197,000.0–373,000.0)	244,000.0 (185,750.0–308,750.0)	0.192
MDW	26.4 (21.3–29.4)	25.0 (21.2–27.8)	0.540
MPV	7.6 (7.1–7.9)	7.5 (7.0–8.0)	0.723
hsCRP, mg/dL	0.8 (0.0–3.2)	0.3 (0.1–1.0)	0.078
PCT, ng/ml	0.5 (0.2–14.4)	0.2 (0.1–0.7)	0.153
D-dimer, ng/ml	940.9 (744.9–1,130.2)	818.3 (444.5–1,465.7)	0.734
LDH, U/L	276.0 (218.8–309.0)	266.0 (216.5–299.5)	0.974
ALT, U/L	16.0 (10.8–24.0)	16.0 (12.0–23.3)	0.697
AST, U/L	29.5 (28.0–33.0)	35.0 (31.0–52.0)	0.117
Cr, mg/dL	0.3 (0.2–0.4)	0.3 (0.2–0.4)	0.640
Na, mmol/L	136.0 (135.0–138.0)	137.0 (136.0–139.0)	0.137
K, mmol/L	4.5 (3.8–4.8)	4.4 (4.0–4.9)	0.800
Glucose, mg/dL	94.0 (81.0–101.5)	94.0 (86.0–108.0)	0.233

ALT, alanine transaminase; AST, aspartate transaminase; COVID-19, coronavirus disease 2019; Cr, creatinine; Hb, hemoglobin; hsCRP, high-sensitivity C-reactive protein; IQR, interquartile range; K, potassium; LDH, lactate dehydrogenase; MDW, monocyte distribution width; MPV, mean platelet volume; NA, sodium; PCT, procalcitonin; WBC, white blood cell. Bold values indicate that the p-value < 0.05.

**TABLE 3:** Comparison of treatment and outcomes of both groups.

Variable median (IQR or %)	COVID-19 with bacterial coinfection (*N* = 24)	COVID-19 (*N* = 137)	*p*-value
**Treatment**			
HFNC oxygen	4 (16.7)	4 (2.9)	**0.019**
Remdesivir	3 (12.5)	2 (1.5)	**0.025**
Paxlovid	0 (0.0)	2 (1.5)	0.687
Dexamethasone	1 (4.2)	21 (15.3)	0.252
IVIG	1 (4.2)	3 (2.2)	0.891
**Outcome**			
Fever duration, days	3.0 (2.0–6.5)	3.0 (2.0–3.0)	0.081
Length of hospital stay, days	5.0 (3.0–7.5)	3.0 (2.0–4.0)	**< 0.001**
ICU admission	6 (25)	18 (13.1)	0.232
Length of ICU stay, days	5.0 (3.0–8.0)	2.0 (1.0–3.0)	**0.007**
Mortality	0	0	–

COVID-19, coronavirus disease 2019; HFNC, high-flow nasal cannula; ICU, intensive care unit; IQR, interquartile range; IVIG, human intravenous immunoglobulin. Bold values indicate that the p-value < 0.05.

Univariable logistic regression analyses showed that fever that persisted more than 3 days before admission [odds ratio (OR), 3.34; 95% confidence interval (CI), 1.03–10.84; *p* = 0.045], abdominal pain (OR, 22.33; 95% CI, 5.37–92.82; *p* < 0.001), diarrhea (OR, 6.81; 95% CI, 2.53–18.38; *p* < 0.001), comorbidity with neurologic diseases (OR, 19.43; 95% CI, 1.93–195.61; *p* = 0.011), PCR cycle threshold value > 20 (OR, 3.62; 95% CI, 1.28–10.23; *p* = 0.015), and hsCRP level > 2 mg/dL (OR, 7.62; 95% CI, 2.67–21.72; *p* < 0.001) were risk factors for bacterial coinfections. After multivariable logistic regression analyses, abdominal pain (OR, 18.87; 95% CI, 2.92–122.16; *p* = 0.002), diarrhea (OR, 7.09; 95% CI, 1.81–27.73; *p* = 0.005), and comorbidity with neurologic diseases (OR, 37.62; 95% CI, 2.45–576.02; *p* = 0.009) remained risk factors for bacterial coinfections in children with COVID-19 (Table 4).

**TABLE 4:** Univariable and multivariable logistic regression analyses of COVID-19 with bacterial coinfections.

	OR	95% CI	*p*-value
**Univariable**			
Age > 3 years	2.29	0.94–5.55	0.068
PCR CT value > 20	3.62	1.28–10.23	**0.015**
Fever duration before admission > 3 days	3.34	1.03–10.84	**0.045**
Abdominal pain	22.33	5.37–92.82	**< 0.001**
Diarrhea	6.81	2.53–18.38	**< 0.001**
Neurologic disease	19.43	1.93–195.61	**0.011**
WBC count > 15,000/μL	4.75	0.99–22.74	0.051
hsCRP > 2 mg/dL	7.62	2.67–21.72	**< 0.001**
**Multivariable**			
Abdominal pain	18.87	2.92–122.16	**0.002**
Diarrhea	7.09	1.81–27.73	**0.005**
Neurologic disease	37.62	2.45–576.02	**0.009**

Variables with p ≤ 0.20 in the univariable analyses were included in the multivariable logistic regression analysis, and the enter method was used to determine baseline risk factors. CI, confidence interval; COVID-19, coronavirus disease 2019; CT, cycle threshold; CRP, C-reactive protein; hsCRP, high-sensitivity C-reactive protein; OR, odds ratio; PCR, polymerase chain reaction; WBC, white blood cell. Bold values indicate that the p-value < 0.05.

## Discussion

This retrospective, observational study was conducted during the first wave of the Omicron outbreak in Taiwan, which revealed that the presence of COVID-19 with bacterial coinfections was not rare in children. Children with COVID-19 with bacterial coinfections often presented with abdominal pain and diarrhea. The rate of comorbidity with neurologic diseases was higher in children with bacterial coinfections. We found that higher cycle threshold values of the PCR test and WBC levels were associated with bacterial coinfections. Our multivariate logistic regression analyses suggested that abdominal pain, diarrhea, and comorbidity with neurologic diseases remained risk factors for bacterial coinfections in children with COVID-19.

A previous study documented that the bacterial coinfection rate of the COVID-19 outpatient population is low (≤ 1%) (11). The incidence rates of community-acquired bacterial coinfections among hospitalized adults with COVID-19 range from 2.5 to 5.1% (11–16). The incidence rate of bacterial coinfections has been documented less often among the pediatric population. A single-center, prospective, observational study performed in India found that 15.03% of pediatric patients with COVID-19 had bacterial coinfections at admission during the non-Omicron period (17). Our study revealed that the incidence rate of bacterial coinfections among hospitalized children with COVID-19 was 14.9% during the Omicron pandemic. Of the hospitalized children with COVID-19, 8.1% had microbiologically confirmed infections.

Genitourinary tract infections were the most common clinical diagnosis, accounting for 57 to 70% of adults with COVID-19 with bacterial coinfections, (15, 18) followed by lower respiratory tract infections (19%), (15) skin and soft tissue infections (1%), (19) and bacteremia (1%) (11, 15, 19). These findings are similar to those of our study, but the rankings were different. During our study, respiratory tract infections (33.3%) were the most common bacterial coinfections. Most were diagnosed according to clinical judgment. Furthermore, most children with COVID-19 with bacterial coinfections had gastrointestinal infections (29.1%), followed by urinary tract infections (20.8%), skin and soft tissue infections (20.8%), and bacteremia (4.1%). These diagnoses were usually proven by the microbiological results. The commonly isolated pathogens of adult COVID-19 patients with bacterial coinfections were *Klebsiella* spp., *E. coli*, *H. influenzae*, *S. pneumoniae*, and *S. aureus* (11, 12, 20). *E. coli* and *Salmonella* spp. were commonly isolated pathogens among this pediatric population, followed by *Campylobacter* spp., *C. difficile*, methicillin-resistant *S. aureus*, and methicillin-sensitive *S. aureus*. These findings indicate that bacterial coinfections might exist at sites other than the lungs in patients with COVID-19 on admission. Therefore, carefully obtaining the patient history, including extrapulmonary symptoms, and performing a targeted physical examination including laboratory tests can help clinicians diagnose bacterial coinfections and administer antibiotics appropriately.

For adults, older age, chronic renal disease, and previous admission to a medical care facility are risk factors for bacterial coinfections (11, 14, 21). Patients with severe and critical clinical presentations after COVID-19 infection are at higher risk for bacterial coinfections (22). A higher percentage of adults with bacterial coinfections received systemic steroids (22). However, unlike adults, age and steroid use are not risk factors for pediatric bacterial coinfections. Although comorbidities such as chronic renal diseases, diabetes, and immunodeficiency disorders are established risk factors for bacterial coinfection in adults with COVID-19 (11, 12), it is currently unclear how comorbidities affect the risk of bacterial coinfection in children with COVID-19. During our study, comorbidity with neurologic diseases were significantly stronger risk factors for bacterial coinfections in children with COVID-19. Among these patients with neurologic diseases, one was epilepsy under medication control, another had Duchenne muscular dystrophy, and the third had spastic quadriplegic cerebral palsy. No significant association was found between bacterial coinfections in children with COVID-19 and other systemic comorbidities.

Fever was more commonly observed in children with COVID-19 during the Omicron wave than during the previous variant waves (7, 23–25). Furthermore, the rate of fever was more than 80% (23, 25). The mean febrile period was less than 2 days during Omicron infection (26). During the current study, we found that children with COVID-19 with fever for more than 3 days before admission might have bacterial coinfections. Unlike the previous variants, Omicron mainly affects the upper respiratory tract. Gastrointestinal symptoms, including vomiting, diarrhea, and abdominal pain, could be present with Omicron infection (9, 23, 25). However, among pediatric patients with the Omicron variant, gastrointestinal symptoms were less common than respiratory symptoms (9, 23, 25). Abdominal pain was observed at a lower rate than other gastrointestinal symptoms (9, 23, 25). During our study, compared with children with only COVID-19, a significantly higher percentage of children with bacterial coinfections presented with abdominal pain (33.3 vs. 2.2%; *p* < 0.001) and diarrhea (41.7 vs. 9.5%; *p* < 0.001). Of eight patients with abdominal pain, three had bacterial gastroenteritis and three had urinary tract infections. Seven of ten patients with diarrhea had enteritis and two had urinary tract infections. Therefore, pediatric COVID-19 patients who present with abdominal pain or diarrhea should be evaluated for bacterial coinfections. Furthermore, urinary tract infections and bacterial enteritis should be included in the differential diagnoses. It is important to consider the possibility of the multisystem inflammatory syndrome in children (MIS-C) in COVID-19 children presenting with abdominal pain and diarrhea. However, in this study, children with abdominal pain and bacterial enteritis did not meet the criteria of MIS-C as they did not exhibit other associated signs. Additionally, none of these patients received immunomodulatory therapy for MIS-C, such as intravenous immunoglobulin (IVIG) or glucocorticoids.

Children may have difficulty explaining their somatic disorders. Therefore, it can be challenging to differentiate children with COVID-19 who may or may not have bacterial coinfections. However, laboratory test results offer clues to which children with COVID-19 also have bacterial coinfections. The WBC, procalcitonin (PCT), and C-reactive protein (CRP) levels are frequently used biomarkers that can differentiate viral and bacterial infections (27). The CRP level is an acute phase reactant that represents the inflammation level and is not specific for diagnosing bacterial infections. Compared with the CRP level, the PCT level is considered a more useful diagnostic biomarker for differentiate between bacterial and viral infections (28). One study indicated that PCT values more than 0.55 ng/ml had 91% sensitivity and 81% specificity for detecting bacterial infections in hospitalized adults with COVID-19 (29). A higher WBC level was observed among children with COVID-19 with bacterial coinfections during our study. A hsCRP level more than 2 mg/dL might be an indicator of bacterial coinfections. However, there was no significant difference in the PCT levels of the two groups. A higher level of viral load is typically associated with increased inflammatory biomarkers. In the current study, higher PCR test cycle threshold values actually indicated lower viral loads. This finding suggests that the higher WBC counts and higher PCR test cycle threshold may reflect an underlying bacterial coinfection.

For adults with COVID-19, bacterial coinfections were associated with worse outcomes. Higher rates of ventilation use, higher mortality rates, and longer hospitalization durations were reported for these patients (12, 20, 30). For children infected with a SARS-CoV-2 variant other than Omicron, the outcomes of bacterial coinfections were similar to those of adults. The rates of mechanical ventilator use, intensive care unit admission, inotropic agent use, and mortality were significantly higher and hospitalization durations were significantly longer among children with COVID-19 with coinfections (17). Fortunately, no deaths were caused by bacterial coinfections among children with COVID-19 in our study during the Omicron pandemic. However, the rates of high-flow nasal cannula oxygen use and remdesivir use were significantly higher in the bacterial coinfection group. Hospital and intensive care unit stays were significantly longer for children with COVID-19 with bacterial coinfections.

There were several limitations to our study. The retrospective design restricted the control of multiple confounders and data collection, and there may have been a selection bias. The enrolled patients were admitted to the hospital via the emergency department. The sample size of this study was small. The bacterial coinfection rate might have been overestimated. The results might not be applicable to the entire pediatric population with COVID-19. This study was conducted at a single medical center. The identification of bacterial coinfections may reflect a site-specific microbiological diagnostic profile. However, this study highlighted the need for clinicians to consider that bacterial coinfections may be present in children with COVID-19. Additionally, the results of this study offer some clues to discovering bacterial coinfections in children affected by COVID-19.

Although infections with the Omicron variant were less severe among children, bacterial coinfections can worsen the progression of disease. Our study provides clinicians with benchmarks for the detection of COVID-19 in children and its possible association with bacterial infections. Hospitalized children with COVID-19 and neurologic diseases who present with abdominal pain or diarrhea are risk for bacterial coinfections. A prolonged fever duration and higher PCR test cycle threshold values, WBC levels, and high-sensitivity CRP levels may be indicators of bacterial coinfections in children with COVID-19. These factors should prompt clinicians to rapidly identify bacterial infections so that appropriate treatment can be provided.

## Data availability statement

The data analyzed in this study is subject to the following licenses/restrictions: The data are not publicly available due to privacy or ethical restrictions. Requests to access these datasets should be directed to Y-LH, codan5230@gmail.com.

## Ethics statement

The studies involving human participants were reviewed and approved by the Institutional Review Board of China Medical University Children’s Hospital (CMUH111-REC3-134). Written informed consent from the participants’ legal guardian/next of kin was not required to participate in this study in accordance with the national legislation and the institutional requirements.

## Author contributions

H-CLai, Y-LH, and H-CLin conceptualized and designed the study, drafted the initial manuscript, and reviewed and revised the manuscript. Y-LH, H-CLai, C-HL, H-MW, J-AC, Y-YL, and Y-TC designed the data collection instruments, collected data, performed the initial analyses, and reviewed and revised the manuscript. Y-LH, H-CLin, and K-PH conceptualized and designed the study, coordinated and supervised data collection, and critically reviewed the manuscript for important intellectual content. All authors approved the final manuscript as submitted and agreed to be accountable for all aspects of the work and approved for publication.

## Footnotes

1
https://www.medcalc.org
