# Supplementary material for: Bacterial coinfections in hospitalized children with COVID-19 during the SARS-CoV-2 Omicron BA.2 variant pandemic in Taiwan
Source: Front Med (Lausanne). 2023 Apr 18;10:1178041. doi: 10.3389/fmed.2023.1178041 (PMC10151712; doi:10.3389/fmed.2023.1178041)
Supplement: Supplementary file 1 [file Table_1.docx]

Supplemental Table 1. Characteristics of confirmed and probable bacterial infections, specimen types, and organisms identified

| Number (%) | Confirmed bacterial infections  N=13 | Probable bacterial infections  N=11 |
| --- | --- | --- |
| **Source and diagnosis** | | |
| Respiratory tract | 1 (7.7) | 7 (63.6) |
| Bronchopneumonia/pneumonia | 0 (0.0) | 6 (54.5) |
| Acute sinusitis | 0 (0.0) | 1 (9.1) |
| Acute otitis media | 1 (7.7) | 0 (0.0) |
| Gastrointestinal tract | 6 (46.2) | 1 (9.1) |
| Bacterial enteritis | 6 (46.2) | 1 (9.1) |
| Urinary tract | 4 (30.8) | 1 (9.1) |
| Renal abscess | 1 (7.7) | 0 (0.0) |
| Acute pyelonephritis | 3 (23.1) | 1 (9.1) |
| Blood stream | 1 (7.7) | 0 (0.0) |
| Sepsis | 1 (7.7) | 0 (0.0) |
| Skin and soft tissue | 1 (7.7) | 2 (18.2) |
| Cellulitis with abscess formation | 1 (7.7) | 0 (0.0) |
| Cellulitis | 0 (0.0) | 2 (18.2) |
| **Specimen type and organisms identified** | | |
| Blood |  |  |
| *Escherichia coli* | 1 (7.7) | - |
| Stool* |  |  |
| *Salmonella spp.* | 2 (15.4) | - |
| *Campylobacter spp.* | 2 (15.4) | - |
| *Clostridium difficile* (toxin A/B) | 3 (23.1) | - |
| Urine |  |  |
| *Escherichia coli* | 4 (30.8) | - |
| Pus |  |  |
| Methicillin-resistant *Staphylococcus aureus* | 1 (7.7) | - |
| Methicillin-sensitive *Staphylococcus aureus* | 1 (7.7) | - |

*One stool contained two pathogens.
